# Morphological and molecular characterization of nematodes in a freshwater fish community from La Mojana, Sucre, Colombia

**DOI:** 10.1017/S003118202610273X

**Published:** 2026-08

**Authors:** Katerin Fuentes-Lopez, Karina Caballero-Gallardo, Robert Poulin, Jerusha Bennett

**Affiliations:** 1 School of Pharmaceutical Sciences, University of Cartagena, Cartagena, Colombia; 2 Department of Zoology, University of Otago, Dunedin, New Zealand

**Keywords:** *Contracaecum*, freshwater fish, helminth, parasite, *Spirocamallanus*

## Abstract

Nematodes constitute one of the most diverse and ecologically significant parasite groups in fishes, yet their diversity and host associations remain poorly documented in tropical freshwater systems. In this study, a community-wide survey of 10 fish species from La Mojana wetland was conducted to assess nematode diversity and host associations. The highest infection indices were recorded in *Pseudoplatystoma magdaleniatum* (Pimelodidae), *Sorubim cuspicaudus* (Sciaenidae) and *Sternopygus macrurus* (Sternopygidae). Morphological examination revealed 2 distinct nematode morphotypes corresponding to larval *Contracaecum* (Anisakidae) and adult camallanids. Molecular analyses based on ITS2, 18S rRNA, 28S rRNA and mitochondrial *rrnS* markers resolved well-supported lineages corresponding to *Contracaecum jorgei* Sardella, Mancini, Salinas, Simões & Luque, 2020 and *Spirocamallanus* sp. The multilocus approach demonstrated the coexistence of phylogenetically distinct nematode groups within the same fish community. Patterns of parasite association revealed a broader host distribution for *Contracaecum*, whereas *Spirocamallanus* showed more restricted occurrence. Coinfections reflected the structural complexity of parasite communities in this tropical system. Because fish represent a primary protein source for local communities in La Mojana, the detection of anisakids raises considerations for food safety and public health. This study provides the first multilocus characterization of nematode diversity and host associations in the region, contributing to the understanding of parasite ecology in tropical freshwater ecosystems.

## Graphical abstract

**Figure:**
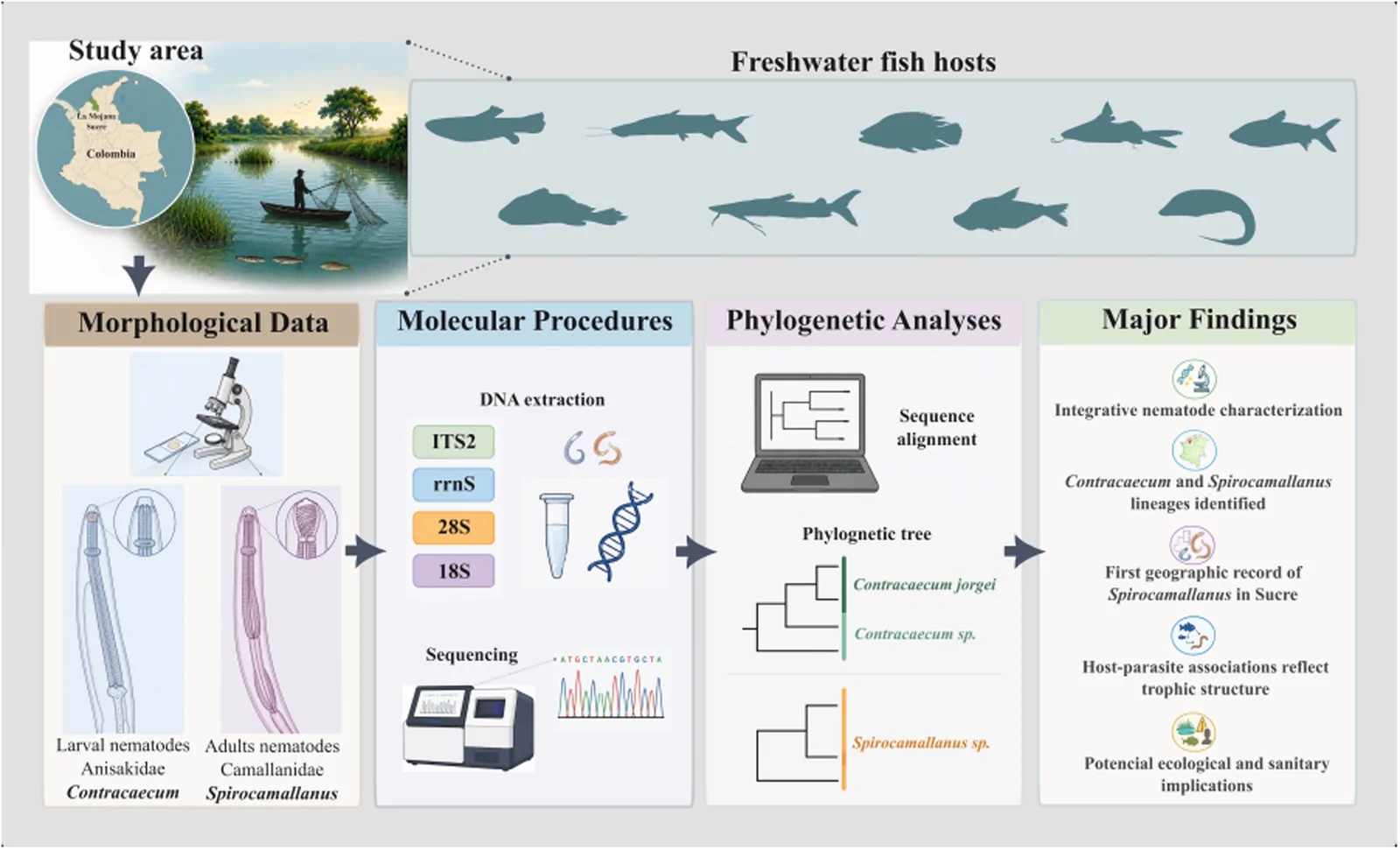

## Introduction

Despite their importance, parasitic nematodes remain undercharacterized, and their role in ecosystem functioning is still poorly understood. Many taxa are undescribed or insufficiently studied, particularly in tropical freshwater systems, where fish-parasitic nematodes represent an especially neglected component of biodiversity (Jezkova et al., 2017; Gendron et al., 2023). In these biodiverse ecosystems, morphologically similar but genetically distinct conspecific nematodes may utilize different transmission routes to reach the same definitive hosts, or different routes to reach different definitive hosts (Bennett et al., 2022; Sures et al., 2025; Viriyautsahakul et al., 2025). In other words, different patterns of host specificity for intermediate hosts might structure assemblages of closely related nematode species. Traditionally, such subtle diversity and life cycle variation may have been missed by studies relying solely on morphological identification (Nadler and Pérez-Ponce de León, 2011). Yet accurate species identification is often critical for mitigating the risk of zoonotic infections (Pérez-Ponce de León and Nadler, 2010).

Among fish-borne nematodes, anisakids are of particular global concern because of their zoonotic significance (Polimeno et al., 2021; Papapostolou et al., 2025). In particular, some species of *Contracaecum* have been recognized for their zoonotic potential, as humans may accidentally ingest larvae through the consumption of raw or undercooked fish, or may develop allergic reactions to parasite-derived antigens. However, zoonotic risk varies among species, highlighting the importance of accurate taxonomic identification when assessing potential risks to human health (Hamouda and Younis, 2022; Kaba et al., 2024). Anisakids normally complete their life cycle in marine mammals or birds as definitive hosts, while fish serve as paratenic hosts for the infective third-stage larvae (L3). Morphological identification of anisakid larvae is extremely difficult because L3 larvae are poorly developed and lack distinctive features. For this reason, molecular markers such as ITS, 12S and 28S, or the mitochondrial genes *cox1* and *cox2* can be helpful tools for accurate identification (Takano and Sata, 2022; Bao et al., 2023; Gu et al., 2024; Yousaf et al., 2025), although they are not without limitations (Ailán-Choke and Pereira, 2021). The use of molecular approaches is increasingly seen as essential to resolve nematode diversity, evolution and host–parasite interactions (Bhat et al., 2022; Do Carmo et al., 2025; Yue et al., 2025).

Within anisakids, *Contracaecum* consists of complexes of sibling species that parasitize piscivorous birds or mammals, particularly birds of the families Pelecanidae, Phalacrocoracidae and Ardeidae (Caffara et al., 2023; Wells, 2023; Garbin et al., 2024b). Their life cycle is complex and not yet fully elucidated for most species, but generally involves eggs released into water by definitive hosts, development to L1–L2 within eggs, ingestion by aquatic invertebrates as first intermediate hosts and transmission to a wide range of fish serving as second intermediate or paratenic hosts. Piscivorous birds and mammals complete the cycle by consuming infected fish (Shamsi, 2019; Bennett et al., 2022; Jawad et al., 2022). *Contracaecum* species, with their often high local diversity within a single community (e.g. Bennett et al., 2022), may display subtle, often cryptic, interspecific variation in life cycle pathways.

Other nematode groups, such as camallanids, are also common parasites of freshwater fishes and may play important ecological roles in aquatic food webs. Members of *Spirocamallanus* (Camallanidae) are intestinal parasites characterized by indirect life cycles involving aquatic invertebrates as intermediate hosts and fish as definitive hosts. Species of *Spirocamallanus* have been reported from a wide range of Neotropical freshwater fishes and are increasingly recognized for their diversity, host associations, infection dynamics and pathological effects in tropical ecosystems (Santana-Piñeros et al., 2017; Neves et al., 2020; Sánchez et al., 2024). However, molecular information remains limited for many species, restricting current understanding of their taxonomy, evolutionary relationships and host specificity.

In Colombia, La Mojana, located in the department of Sucre, is a wetland region recognized for its rich biodiversity and abundant water and soil resources. The region faces high poverty levels and increasing environmental pressures, such as mining activities and the intensive use of pesticides in agriculture (Baleta-Anaya et al., 2022; Romero-Suárez et al., 2022; Marrugo-Negrete et al., 2024). Fish represent a primary source of animal protein for local communities in La Mojana, where fishing constitutes a key subsistence activity. Consequently, understanding parasite diversity in edible fish is not only relevant from an ecological perspective but also from a food security and public health standpoint (Baleta-Anaya et al., 2022). In addition, the region is affected by environmental contamination associated with artisanal gold mining (Espitia-Pérez et al., 2025). *Contracaecum* larvae have been reported in freshwater fish from La Mojana (Wadnípar-Cano, 2013), where environmental degradation and water contamination add to existing ecological pressures. These nematodes link fish populations with piscivorous birds but may also impair fish health, reduce the quality of fish for consumption and represent a potential risk of zoonosis to local communities (Fuentes-Lopez et al., 2025). While a few records exist (Wadnípar-Cano, 2013; Fuentes-Lopez et al., 2025), the morphological and molecular diversity of *Contracaecum* species and their transmission pathways through fish hosts, including species widely consumed by local people, remain unknown.

Knowledge about the diversity, host range, morphological traits and genetic identity of nematodes infecting freshwater fishes in La Mojana remains limited. In this context, the present study aimed to characterize nematodes associated with a freshwater fish community from La Mojana, through an integrative morphological and molecular approach. By investigating multiple fish species and different nematode taxa, this study provides an initial step towards clarifying parasite diversity, host associations and some trophic transmission pathways of these nematodes in tropical inland waters.

## Materials and methods

### Specimen collection

A total of 50 specimens of 10 freshwater fish species were collected in April 2025 from San Jorge River basin in San Marcos, within the La Mojana wetland region, Sucre (Colombia) (8°35′06″ N, 75°07′16.39″ W). Local fishermen assisted in the captures using cast nets and trammel nets. Fish specimens were stored on ice until *in situ* processing. Five specimens of 10 fish species were analysed (Table 1): *Hoplias malabaricus* (trophic position *n* = 4.5), *Plagioscion surinamensis* (trophic position *n* = 4.5), *Pseudoplatystoma magdaleniatum* (trophic position *n* = 4.3), *Sorubim cuspicaudus* (trophic position *n* = 4.3), *Cynopotamus magdalenae* (trophic position *n* = 4.2), *Polydactylus virginicus* (trophic position *n* = 3.7), *Trachelyopterus insignis* (trophic position *n* = 3.5), *Caquetaia kraussii* (trophic position *n* = 3.4), *Sternopygus macrurus* (trophic position *n* = 3.2) and *Megaleporinus muyscorum* (trophic position *n* = 2.6). Trophic positions are from FishBase (https://fishbase.se/search.php) and range from apex piscivores at 4.5+ down to herbivores at 2.0. Each fish was measured for total length (cm) and body weight (g), and then dissected for visual examination of internal organs. All nematodes encountered were collected, counted and preserved in 70% ethanol for whole-mount preparations or in 96% ethanol and stored at −20 °C for molecular analyses (Fuentes-Lopez et al., 2025). Mean parasite descriptors were calculated following Bush et al. (1997). Mean abundance represented the average number of parasites per examined host, including both infected and uninfected individuals, whereas mean intensity was defined as the average number of parasites per infected host. These descriptors were calculated separately for each host species.

**Table 1.** Fish species examined in this study, with family, sample size (*n*), trophic level, biometric data (total length, weight), feeding habit and infection parameters Table 1 long description.

Common name	Scientific name	Family	Feeding habit	Sample size (n)	Trophic level	Mean total length (cm)	Mean total weight (g)	Number of parasites	Mean abundance ± SEM	Mean intensity ± SEM
Moncholo	*Hoplias malabaricus*^b^	Erythrinidae	Carnivore (piscivore)	5	4.5	31.6	337.6	21	4.2 ± 1.4	5.3 ± 1.1
Pacora	*Plagioscion surinamensis*^b^	Sciaenidae	Carnivore (piscivore)	5	4.5	28.1	175.2	5	1.0 ± 0.0	1.0 ± 0.0
Bagre blanquillo	*Sorubim cuspicaudus*^b^	Sciaenidae	Carnivore (piscivore)	5	4.3	47.9	562.8	77	15.4 ± 10.1	15.4 ± 10.1
Bagre rayado	*Pseudoplatystoma magdaleniatum*^b^	Pimelodidae	Carnivore (piscivore)	5	4.3	49.0	782.4	88	17.6 ± 4.3	17.6 ± 4.3
Chango	*Cynopotamus magdalenae*	Characidae	Carnivore (piscivore)	5	4.2	26.8	179.6	13	2.6 ± 0.7	3.3 ± 0.3
Barbul	*Polydactylus virginicus*^b^	Polynemidae	Carnivore (piscivore)	5	3.7	24.7	115.6	5	1.0 ± 0.3	1.3 ± 0.3
Cachegua	*Trachelyopterus insignis*^c^	Auchenipteridae	Carnivore (piscivore)	5	3.5	20.2	104.0	17	3.4 ± 1.1	4.3 ± 1.0
Mojarra Amarilla	*Caquetaia kraussii*^b^	Cichlidae	Carnivore (piscivore)	5	3.4	21.9	156.8	15	3.0 ± 1.2	3.8 ± 1.3
Mayupa	*Sternopygus macrurus*^c^	Sternopygidae	Carnivore (piscivore)	5	3.2	70.4	686.0	37	7.4 ± 3.7	7.4 ± 3.7
Comelón	*Megaleporinus muyscorum*^b^	Anostomidae	Omnivores	5	2.6	33.5	344.4	11^a^	2.2 ± 0.7	2.8 ± 0.6

a *Notes:* This number includes *Contracaecum* and *Spirocamallanus*.

b Fish species of major commercial importance and commonly consumed by local communities in La Mojana, Sucre, Colombia.

c Fish species consumed less frequently by local communities.

*Source:* Wadnípar-Cano, 2013; Baleta-Anaya et al., 2022.

### Morphological data

Nematodes intended for morphological analyses were cleared in 100% lactophenol for approximately 2 h prior to microscopic examination. Specimens were observed under an Olympus BX51 compound microscope equipped with a DP25 digital camera, and measurements were taken from captured images using ImageJ software (Wayne Rasband, NIH, USA). Identification was carried out to the lowest possible taxonomic level based on the diagnostic characters provided in standard morphological keys (Anderson, 2000) and original species descriptions.

### Molecular procedures

A total of 51 specimens, selected from 289 collected nematodes, were used for molecular analyses to represent the observed morphological diversity and, whenever possible, to include individuals recovered from all fish species. Selection was designed to encompass hosts from different trophic levels, including several carnivorous species and fishes of commercial importance in La Mojana region. This subset comprised specimens morphologically identified as *Contracaecum* as well as a few specimens identified as camallanids.

Genomic DNA was extracted using the DNeasy® Blood & Tissue Kit (Qiagen, Hilden, Germany), following the manufacturer’s protocol. Targeted molecular markers included the internal transcribed spacer region 2 (ITS2), partial small and large ribosomal subunits (18S and 28S, respectively) and partial mitochondrial small subunit rRNA gene (*rrnS*). The full set of markers could not be used for both *Contracaecum* and camallanid specimens, due to amplification failure or, in the case of camallanids, an insufficient number of available specimens.

Polymerase chain reaction (PCR) amplifications for the ITS2, 18S rRNA and 28S rRNA genes were performed in a 10 µL reaction volume containing 6.64 µL nuclease-free water, 2 µL MyTaq™ Red 5× Reaction Buffer (Bioline), 0.4 µL of each primer, 0.12 µL bovine serum albumin (BSA; New England Biolabs), 0.04 µL MyTaq™ DNA polymerase (Bioline) and 0.4 µL extracted DNA. For ITS2 (target base pairs (bp) ∼500), we used primers SS2 and NC2 (Shamsi and Suthar, 2016) under the following PCR conditions of 94°C for 5 min; 30 cycles of 94°C for 30 s, 55°C for 30 s and 72°C for 30 s; followed by a final extension at 72°C for 5 min. For the 18S rRNA gene (∼ 900 bp), primers Nem18SF and Nem18SR (Wood et al., 2013) were used under the following PCR conditions: 94°C for 5 min, followed by 35 cycles of 94°C for 30 s, 54°C for 30 s and 72°C for 1 min and a final extension at 72°C for 10 min. For the 28S rRNA gene (∼800–900 bp), primers T16 and T30 (Harper and Saunders, 2001) were used under the following PCR conditions: 94°C for 5 min, followed by 30 cycles of 94°C for 30 s, 45°C for 30 s and 72°C for 2 min, with a final extension at 72°C for 7 min. For the *rrnS* gene (∼485 bp), PCR amplifications were performed in a 25 µL reaction volume containing 5 µL DNA (10 ng/µL), 1 µL of each primer (10 µM), 1 µL GC enhancer and 12.5 µL AmpliTaq Gold™ 360 Master Mix (Thermo Fisher Scientific, Waltham, MA, USA). Primers MH3 and MH4.5 were used for amplification (D’amelio et al., 2000; Vipindas et al., 2020); PCR conditions included an initial denaturation at 95°C for 10 min, followed by 35 cycles of 95°C for 15 s, 52.4°C for 30 s and 72°C for 1 min, with a final extension at 72°C for 7 min.

Positive PCR products were purified using EXOSAP-IT™ Express PCR Product Cleanup Reagent (USB Corporation, Cleveland, OH, USA), following manufacturer instructions. Purified amplicons were sequenced by the Genetic Analysis Service, Department of Anatomy, University of Otago (Dunedin, New Zealand), using the same forward primers employed for amplification.

### Phylogenetic analyses

Chromatograms were visually inspected, edited and trimmed in Geneious Prime® v21.0.7. Forward reads were used for sequence editing and subsequent phylogenetic analyses. Edited sequences were compared against reference sequences deposited in GenBank using the BLASTn algorithm (https://blast.ncbi.nlm.nih.gov) to guide the selection of taxa for subsequent phylogenetic analyses. Representative reference sequences showing the highest BLAST similarity and representing the known phylogenetic diversity of the target taxa were selected as the ingroup. Outgroup taxa were selected from closely related nematode lineages based on previously published phylogenetic studies (e.g. Bennett et al., 2022) and were used to root each phylogenetic tree. Sequence alignments were generated for each genetic marker using the MUSCLE algorithm with default parameters implemented in MEGA v12.1.2. (Kumar et al., 2024). Alignments were manually inspected and trimmed to remove poorly aligned regions and ambiguous positions. Pairwise genetic distances were calculated for the 28S rRNA dataset, with divergence estimated using the p-distance model with pairwise deletion of gaps and missing data.

Bayesian phylogenetic analyses were conducted in MrBayes v3.2, implemented through the CIPRES Science Gateway (Miller et al., 2010), using a Markov Chain Monte Carlo approach with 2 independent runs, each consisting of 1 cold and 3 heated chains. We estimated a model of evolution for each gene using JModelTest2 v2.1.6, restricted to models compatible with MrBayes using Akaike Information Criteria (Darriba et al., 2012). The resulting models were selected as follows: ITS2 and *rrnS* = HYK + I + G, 18S (*Contracaecum*) = GTR + I, 18S (*Spirocallamanus*) = GTR + I + G, 28S = HYK + G. Each analysis was run for 10 000 000 generations, with trees sampled every 10 000 generations. The heating parameter was set to 0.02 to improve chain mixing. Convergence was assessed by examining the average standard deviation of split frequencies and potential scale reduction factors from CIPRES output files. The first 25% of sampled trees were discarded as burn-in, and a 50% majority-rule consensus tree was generated. Nodal support was evaluated using Bayesian posterior probabilities (BPPs), with values >0.95 considered strong support (Ronquist et al., 2012). Final consensus trees were visualized and edited in FigTree v1.4.4 and Inkscape v1.4.2.

## Results

### Host biometric characteristics

The biometric characteristics of host species, including family, sample size (*n*), trophic level, total length, body weight, feeding habit and the number of parasite taxa recovered, are summarized in Table 1. A total of 50 fish were examined for parasite recovery, yielding 289 nematodes, of which 51 individuals were selected for genetic analyses. Mean parasite abundance varied among host species, with the highest values recorded in *P. magdaleniatum* (17.6 ± 4.3 parasites per fish), followed by *S. cuspicaudus* (15.4 ± 10.1) and *S. macrurus* (7.4 ± 3.7). In contrast, the lowest abundances were observed in *P. surinamensis* (1.0 ± 0.0), *P. virginicus* (1.0 ± 0.32) and *C. magdalenae* (2.6 ± 0.7) (Table 1).

### Morphological data

**Figure 1.**
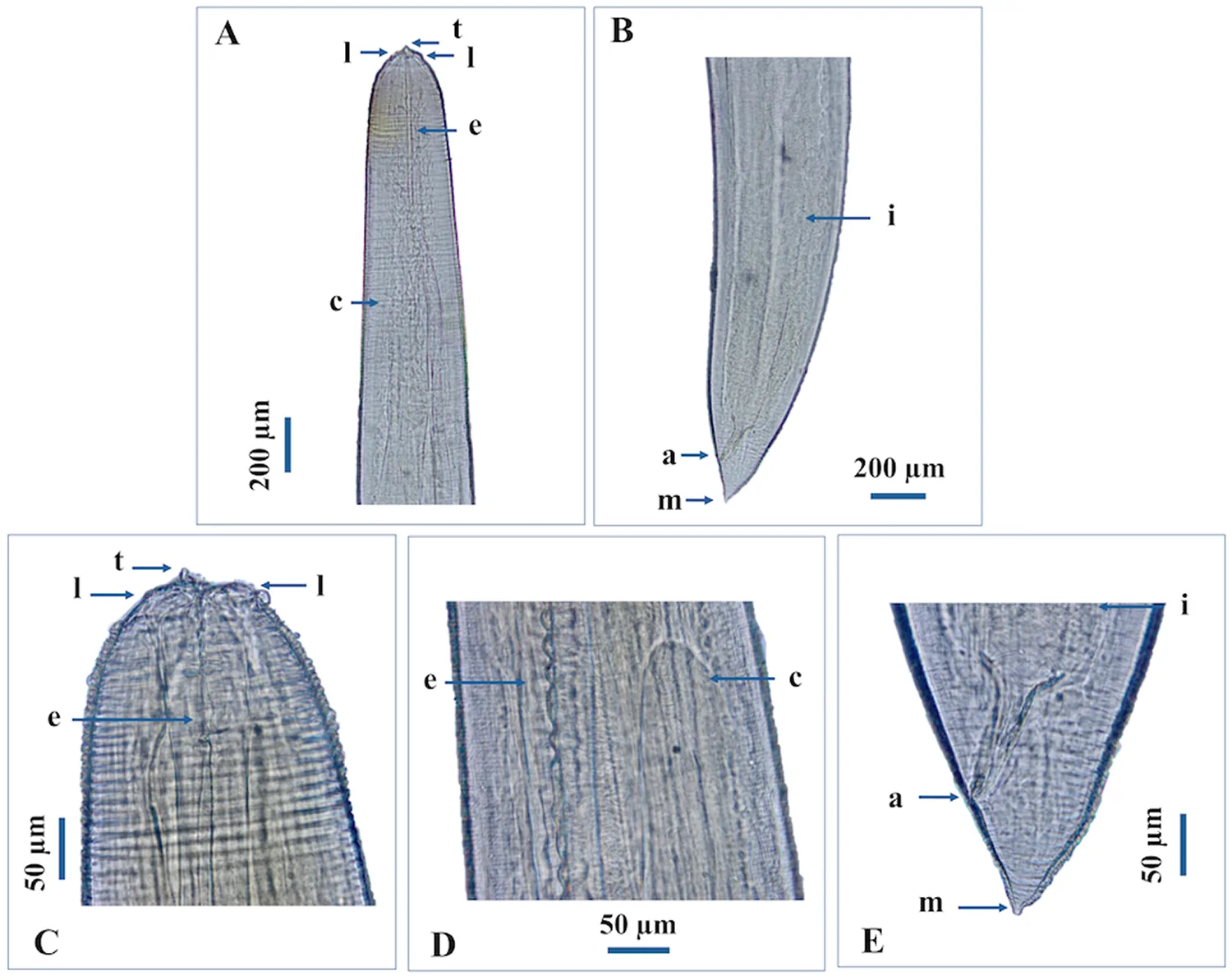
Light micrographs of *Contracaecum* sp. larvae recovered from the freshwater fish *S. cuspicaudus*. (A) Anterior region showing the lips (l), tooth (t) and oesophagus (e) (scale bar = 200 µm). (B) Posterior region showing the intestine (i), anus (a) and terminal mucron (m) (scale bar = 200 µm). (C) Detail of the anterior region showing the lips (l), tooth (t), and oesophagus (e) (scale bar = 50 µm). (D) Midbody region showing the cecum (c) and oesophagus (e) (scale bar = 50 µm). (E) Detail of the posterior region showing the intestine (i), anus (a) and terminal mucron (m) (scale bar = 50 µm).

**Figure 2.**
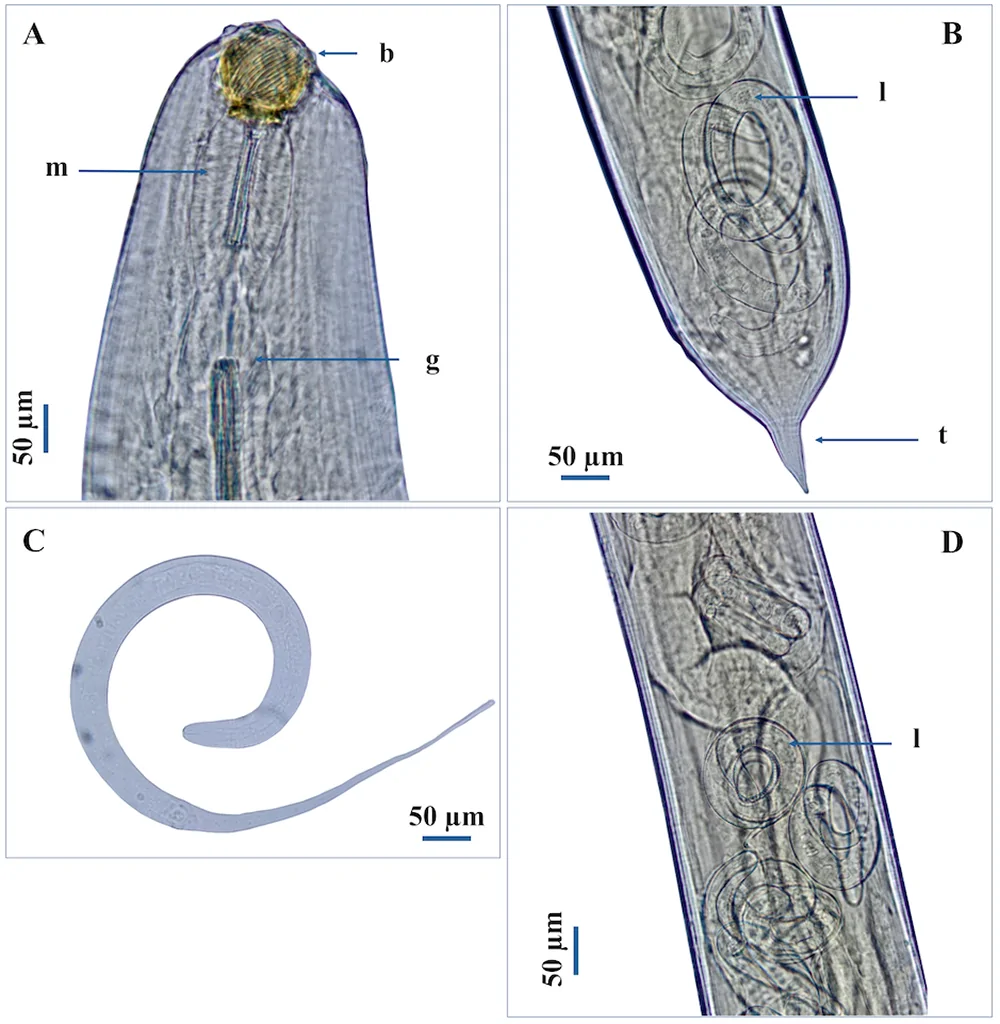
Light micrographs of female specimens of *Spirocamallanus* sp. recovered from the freshwater fish *Megaleporinus muyscorum*. Scale bars = 50 µm. (A) Anterior region showing the buccal capsule (b), muscular oesophagus (m), and glandular oesophagus (g). (B) Posterior region of a gravid female showing first-stage larvae (l) within the uterus and the tail (t). (C) First-stage larva (L1). (D) Midbody region of the female showing first-stage larvae (l) within the uterus.

Morphological examination of the nematodes obtained from fish hosts (Figure S1) revealed the presence of 2 clearly differentiated morphotypes among the examined specimens (Figures 1,2). A total of 289 nematodes were recovered, of which 285 were morphologically identified as *Contracaecum* and 4 as *Spirocamallanus*. All fish species were parasitized by *Contracaecum* third-stage larvae (L3), showing diagnostic characteristics of the genus (family Anisakidae) (Figure S2). The anterior extremity presented 3 lips, a prominent larval tooth, and a well-developed oesophagus. The midbody region showed an intestinal cecum, whereas the posterior extremity displayed the intestine, a distinct anus and a terminal mucron. These features are consistent with *Contracaecum* sp. L3 (Figure 1).

In contrast, adult nematodes recovered from *M. muyscorum* exhibited morphological characteristics consistent with *Spirocamallanus* sp. (Figure S3), including a well-defined sclerotized buccal capsule and a robust body morphology, both typical of adult camallanid nematodes. The anterior region showed a well-developed buccal capsule, followed by a muscular oesophagus and a posterior glandular oesophagus. Gravid female specimens contained numerous first-stage larvae (L1) within the uterus, indicating larviparous reproduction, a characteristic feature of adult females of *Spirocamallanus* (Figure 2).

### Molecular and phylogenetic analyses

**Figure 3.**
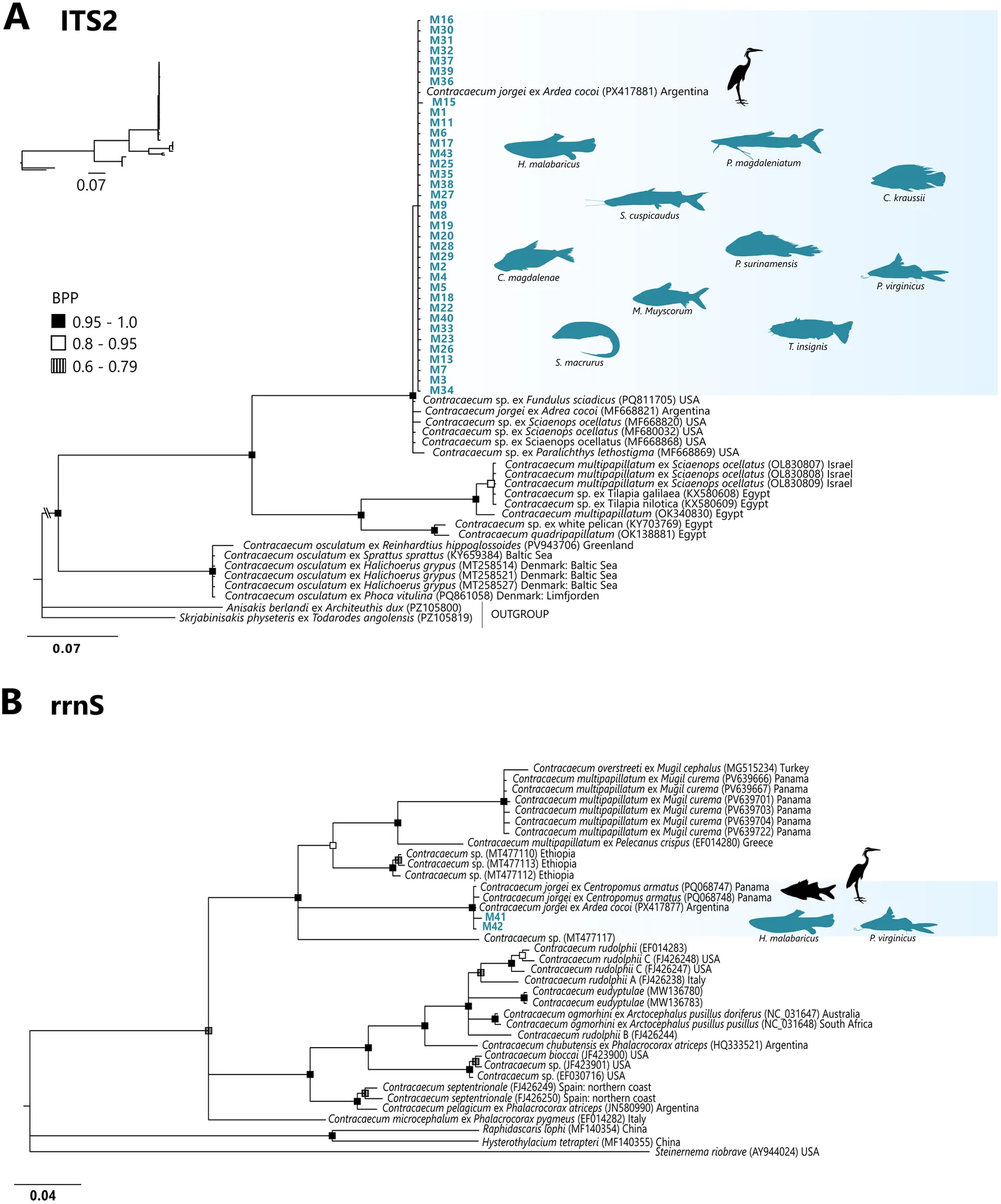
Bayesian inference phylogenetic trees reconstructed separately using 1 of 2 genetic markers: (A) ITS2 and (B) mitochondrial *rrnS*. Specimens analysed in the present study are highlighted in blue, with their host species indicated next to the sequences. Bayesian posterior probabilities (BPP) are indicated at the nodes. Scale bars represent the number of nucleotide substitutions per site. Figure 3 long description.

**Figure 4.**
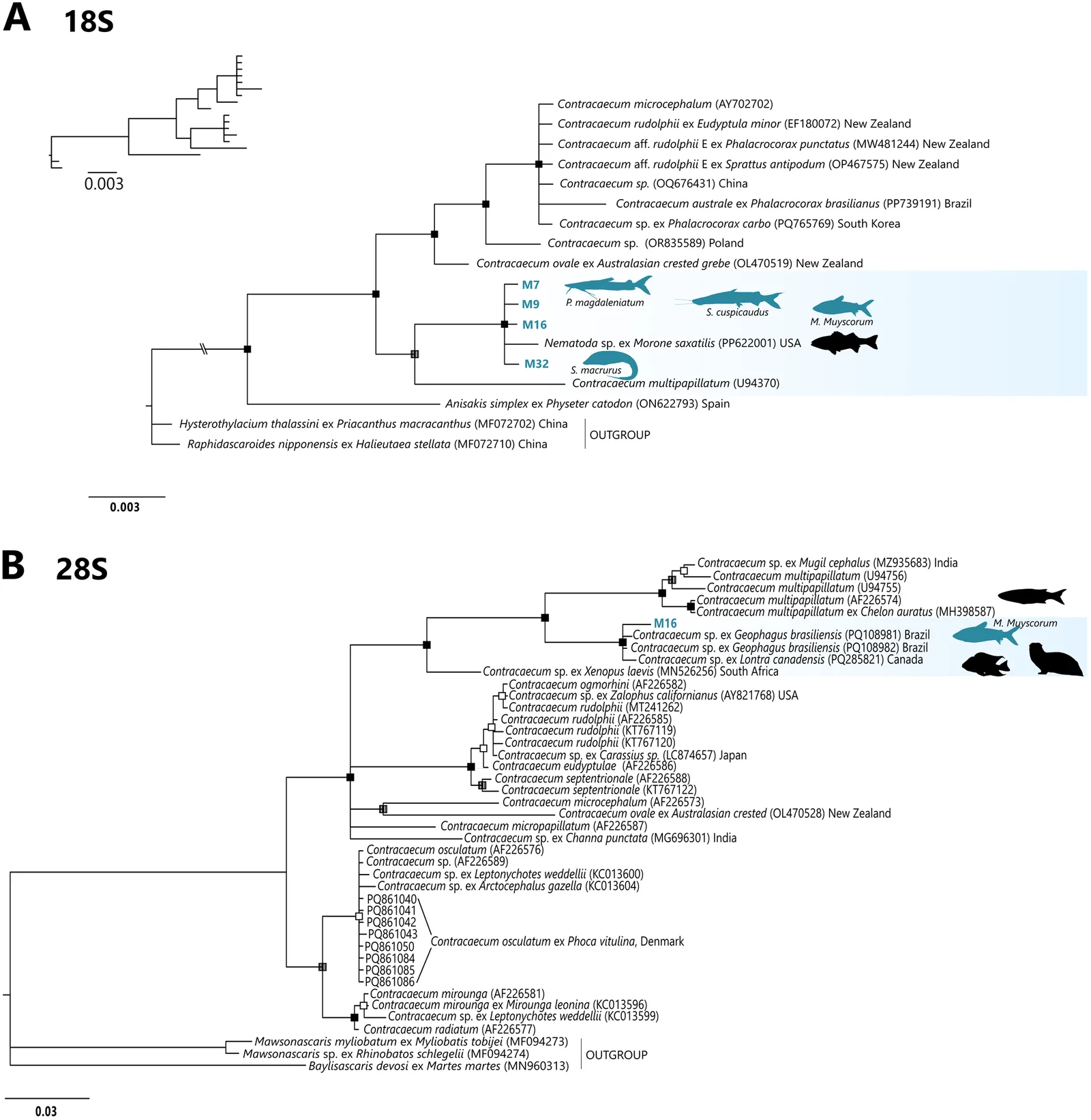
Bayesian inference phylogenetic trees reconstructed separately using 1 of 2 genetic markers: (A) nuclear 18S rRNA, and (B) nuclear 28S rRNA. Specimens analysed in the present study are highlighted in blue, with their host species indicated next to the sequences. Bayesian posterior probabilities (BPPs) are indicated at the nodes. Scale bars represent the number of nucleotide substitutions per site. Figure 4 long description.

**Figure 5.**
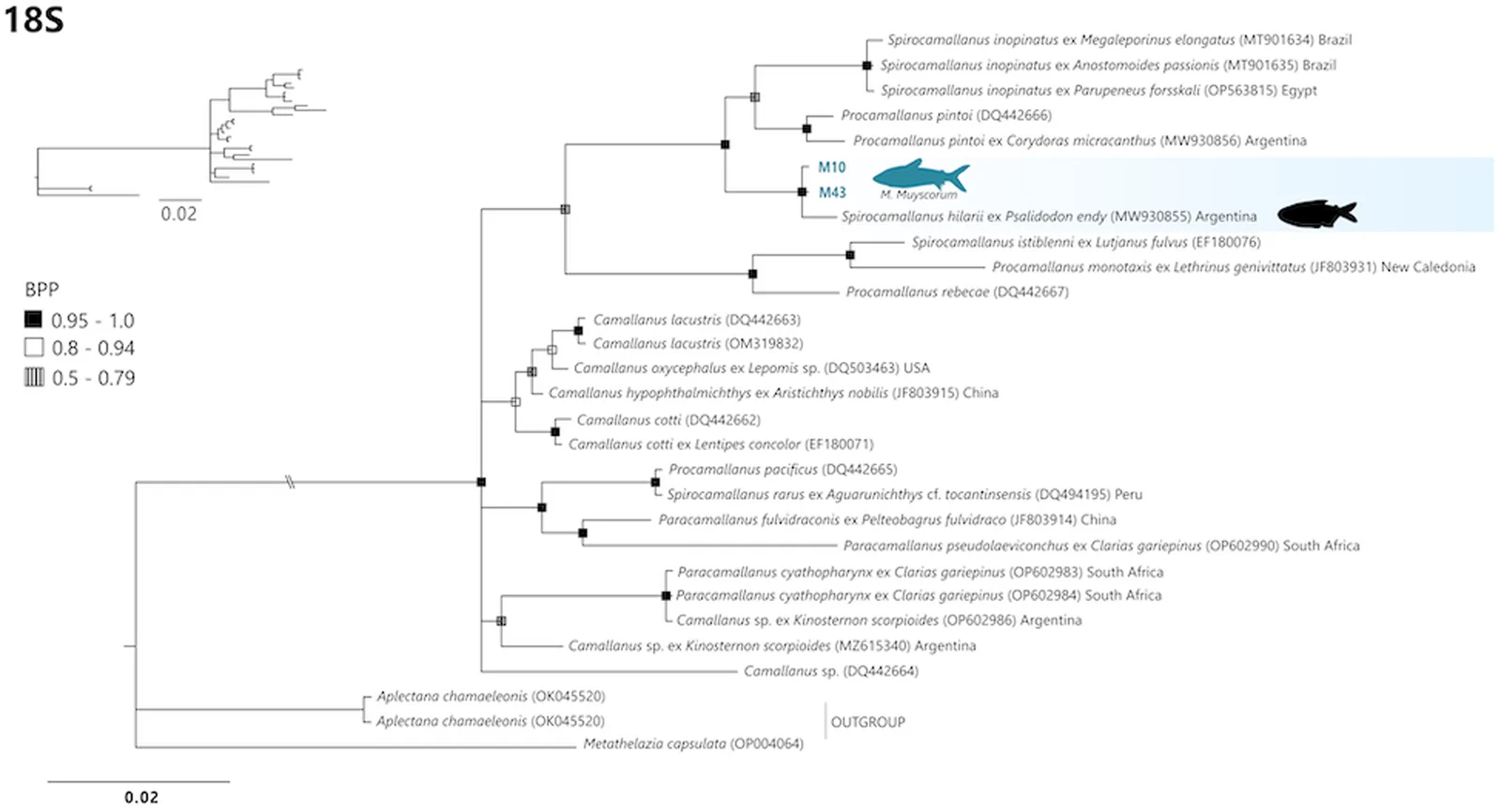
Bayesian inference phylogenetic tree reconstructed using the nuclear 18S rRNA marker for *Spirocamallanus* specimens. Specimens analysed in the present study are highlighted in blue, with their host species indicated next to the sequences. Bayesian posterior probabilities (BPPs) are indicated at the nodes. Scale bar represents the number of nucleotide substitutions per site. Figure 5 long description.

A total of 51 sequences were obtained for molecular analyses, including 37 ITS2, 6 18S, 6 28S and 2 *rrnS* sequences. Of these, 47 sequences corresponded to *Contracaecum* and 4 to *Spirocamallanus* according to BLAST results, supporting our morphological identifications. Sequences obtained from all examined freshwater fish hosts grouped together with high BPP support, indicating genetic similarity among specimens collected from different host species (Figures 3**–**5). The sequences generated in this study were submitted to GenBank, and accession numbers are provided in Table S1.

Phylogenetic reconstruction based on ITS2 (Figure 3A) and mitochondrial *rrnS* (Figure 3B) sequences showed that our specimens formed a well-supported clade within *Contracaecum jorgei* Sardella, Mancini, Salinas, Simões & Luque, 2020, previously isolated from *Ardea cocoi* in Argentina (Garbin et al., 2026), with sequence identities ranging from 95% to 100%. This clade was also closely related to *Contracaecum* sp. previously reported from *Fundulus sciadicus* in the USA. The clade containing our specimens was clearly separated from other congeners, including, among others, the *Contracaecum multipapillatum* species complex and *Contracaecum osculatum* (Rudolphi, 1802) Baylis, 1920. Within both the ITS2 dataset and *rrnS* dataset, our samples exhibited low intraspecific genetic variation (0.21%), indicating high genetic homogeneity among specimens. The mean genetic divergence between our samples and *C. jorgei* was also low (ITS2: 0.23%; *rrnS*: 0.28%), while ITS2 divergence relative to the closely related clade containing *Contracaecum* sp. ex *Fundulus sciadicus* and *Sciaenops ocellatus* ranged around 0.81%. In contrast, substantially higher divergences (14–33%) were observed for both markers between our samples and other *Contracaecum* lineages, supporting the placement of the present specimens within the *C. jorgei* lineage.

The 18S rRNA phylogeny placed specimens recovered from *P. magdaleniatum, S. cuspicaudus, M. muyscorum* and *S. macrurus* within a well-supported clade corresponding to *Contracaecum* sp. (Figure 4A), with sequence identities ranging from 95% to 100%. These sequences clustered closely with *C. multipapillatum* deposited in GenBank under accession number U94370 and were positioned near other congeners, including *C. australe, C. rudolphii, C. microcephalum* and *C. ovale*. For the 18S dataset, our samples showed no intraspecific variation. The lowest mean genetic divergence was observed between samples from this study and *Nematoda* sp. ex *Morone saxatilis* (0.13%). Divergence between our samples and other *Contracaecum* lineages ranged from 0.99% to 1.33%, including *C. ovale* (0.99%), the *C. rudolphii/C. australe/C. microcephalum* complex (1.17%) and *C. multipapillatum* (1.33%). These low divergence values reflect the conserved nature of the 18S marker and its limited resolution for species-level discrimination within *Contracaecum*. Similarly, the 28S rRNA phylogeny placed the specimen recovered from *M. muyscorum* within a well-supported clade of *Contracaecum* sp. (Figure 4B). This sequence showed more than 95% identity with *C. multipapillatum* (sensu lato). For the 28S dataset, our single sample had its lowest mean genetic divergence with the clade containing *Contracaecum* sp. ex *Geophagus brasiliensis* (1.06%). In contrast, substantially higher divergences were observed relative to the *C. multipapillatum* complex (9.18%), *C. rudolphii*-related lineages (12.75%) and the *C. osculatum* complex (12.97%).

Phylogenetic reconstruction based on the 18S rRNA marker placed the second nematode species recovered from *M. muyscorum* within a well-supported clade corresponding to *Spirocamallanus* (Figure 5). The sequences obtained in this study formed a distinct subclade closely related to *Spirocamallanus hilarii* (Vaz & Pereira, 1934), previously reported from *Psalidodon endy* in Argentina and deposited in GenBank under accession number MW930855. This relationship suggests close affinity among South American representatives of *Spirocamallanus*. The clade containing our sequences was clearly separated from other camallanid genera, including *Procamallanus, Camallanus* and *Paracamallanus*, supporting the taxonomic placement of the nematodes recovered from *M. muyscorum.* For the 18S dataset of *Spirocamallanus*, our samples were genetically identical, showing no intraspecific variation. The lowest mean genetic divergence was observed between samples from our study and *S. hilarii* (0.37%). Higher divergences were observed relative to *Procamallanus pintoi* (1.70%), *Spirocamallanus inopinatus* (2.32%) and other related camallanid lineages (5.28%). These results support the placement of our samples within, or very close to, the *S. hilarii* lineage.

In contrast, analyses based on the 28S marker yielded a different pattern. Pairwise genetic distances showed that specimens from *M. muyscorum* differed by only 2.5% from *Procamallanus neocaballeroi*, whereas divergence from *Spirocamallanus inopinatus* exceeded 18%. These results indicate closer molecular affinity with *Procamallanus* sp. than with currently available *Spirocamallanus* reference sequences (Table S2). However, morphological traits of the recovered specimens were consistent with *Spirocamallanus*.

## Discussion

Parasite communities in tropical freshwater ecosystems remain insufficiently characterized despite their ecological and human health relevance and sensitivity to environmental change. Environmental conditions, host diversity and trophic interactions can strongly influence the maintenance and transmission of helminths, particularly those with complex life cycles (Scott, 2023). Our findings of nematodes across multiple fish hosts highlight the wide circulation of these parasites within the freshwater fish community of La Mojana. The occurrence of anisakid larvae in several carnivorous and omnivorous fish species supports the role of these hosts in trophic transmission pathways involving piscivorous vertebrates as definitive hosts, as previously suggested for *Contracaecum* sp. in aquatic ecosystems (Shamsi, 2019; Jawad et al., 2022). In addition, the identification of *Spirocamallanus* expands current knowledge of camallanid nematodes in Colombian freshwater fishes and reinforces the importance of integrative morphological and molecular approaches to improve the taxonomic resolution and understanding of host associations in Neotropical fish parasites (Santana-Piñeros et al., 2017). Nevertheless, the limited number of fish examined and the uneven geographic coverage currently available for La Mojana suggest that the actual diversity and distribution of nematode taxa in this tropical wetland system remain underestimated.

Morphological examination identified 2 nematode taxa, including L3 larvae of *Contracaecum* sp. and adult *Spirocamallanus* sp. The widespread occurrence of *Contracaecum* sp. in La Mojana is consistent with previous records linking anisakid prevalence to eutrophication and high organic matter in floodplain systems (Olivero-Verbel and Caballero-Gallardo, 2013; Wadnípar-Cano, 2013; Fuentes-Lopez et al., 2025). Similar patterns have been documented in other tropical freshwater environments (Abd-Elrahman et al., 2023; Aqeele et al., 2024). The present finding represents the first locality record of *Spirocamallanus* in the department of Sucre, expanding the known distribution of this taxon in Colombia. Camallanids have been reported from the Sierra Nevada de Santa Marta, where they were recovered from *Trichomycterus nigromaculatus* (Toncel-Palencia and García-Alzate, 2024), and in the Orinoquía region where *Procamallanus* (*Spirocamallanus*) cf. *pintoi* was found in *Corydoras metae* (Siluriformes: Callichthyidae) (Santana-Piñeros et al., 2017). Previous reports from neighbouring South American countries suggest the wide distribution of camallanid nematodes among Neotropical fishes (Silva et al., 2026). For example, *Procamallanus* (*Spirocamallanus*) *pintoi* has been reported from species of *Corydoras* in Peru (Sánchez et al., 2024; Murrieta Morey et al., 2025). The high hydrological connectivity and increasing environmental disturbance in Neotropical systems may facilitate parasite dispersal and host switching among aquatic hosts (Lima-Corrêa et al., 2023).

Our integrative morphological and multilocus molecular framework provided taxonomic identification of the recovered nematodes, allowing species-level resolution for *Contracaecum jorgei*. Mitochondrial *rrnS* and nuclear ITS2 sequences showed high similarity to *C. jorgei* in GenBank across all host species examined, consistent with previous molecular diagnoses of this species in Argentina, Brazil and Panama (Garbin et al., 2024a; Duarte et al., 2025; González-Serrano et al., 2025). *Contracaecum jorgei* has been reported from freshwater fishes along the Magdalena River in Colombia (Rave et al., 2026). This record is particularly relevant because the Magdalena River system is hydrologically connected with La Mojana region through a network of rivers and wetlands, facilitating the movement of aquatic organisms and potentially their parasites. Moreover, anthropogenic activities occurring along the Magdalena basin may influence downstream aquatic environments, including water bodies associated with La Mojana (Tejeda-Benitez et al., 2016; Gordon-Morante and Marrugo-Negrete, 2018; Tejeda-Benítez et al., 2023). *Contracaecum* sp. exhibit a broader host distribution, consistent with their heteroxenous life cycle involving piscivorous birds as definitive hosts and fish as intermediate or paratenic hosts (Bennett et al., 2022; Caffara et al., 2023).

Phylogenetic analyses based on 18S and 28S rRNA further revealed the presence of *C. multipapillatum* and *Spirocamallanus* sp. in *Megaleporinus muyscorum*, although species-level identification based on 28S rRNA must be regarded with caution. *Spirocamallanus* has been previously recorded in Colombia, including in *Trichomycterus nigromaculatus* from the Caribbean region (Toncel-Palencia and García-Alzate, 2024) and in ornamental species such as *Corydoras metae* (Santana-Piñeros et al., 2017). Genetic distance analyses based on ITS2, *rrnS*, 18S and 28S showed low variation among the Colombian isolates obtained here and the lowest divergences relative to *C. jorgei* and *S. hilarii*-related lineages, whereas substantially higher divergences were observed relative to other congeners and species complexes. The higher resolution provided by ITS2 and *rrnS*, together with the concordance among phylogenetic analyses across markers, supports the placement of the Colombian specimens within, or closely related to, the *C. jorgei* and *S. hilarii* lineages.

Traditionally, *Spirocamallanus* has been separated from *Procamallanus* by buccal capsule morphology, particularly the presence of spiral ridges on the inner capsule surface, a feature observed in the specimens examined here and supporting their provisional assignment to *Spirocamallanus*. However, recent phylogenetic studies have questioned the reliability of these characters alone, indicating that morphology-based diagnoses may not always reflect evolutionary relationships. As a result, the taxonomic status of *Spirocamallanus* remains debated, although current evidence generally supports its recognition as a distinct genus pending broader systematic revision (Ailán-Choke and Pereira, 2021). Although the morphological characters observed, particularly those of the buccal capsule, were consistent with *Spirocamallanus*, the 28S sequences showed higher similarity to *Procamallanus*. This incongruence may reflect the limited availability of comparative sequences, unresolved taxonomic boundaries between both groups, or inaccuracies in publicly available database records.

The detection of *Spirocamallanus* sp. closely related to *S. hilarii* exclusively in *M. muyscorum*, an omnivorous species (Mosquera-Ramos and Gómez-Ramírez, 2024), suggests that feeding ecology may influence exposure to infective stages. Camallanids typically use aquatic invertebrates as intermediate hosts, and omnivorous or benthic-feeding fishes may have a higher probability of ingesting infected copepods or insect larvae (Iyaji and Yaro, 2016; Murrieta Morey et al., 2025). In contrast to *Contracaecum*, whose adult stages occur in piscivorous birds, several species of *Spirocamallanus* complete their development as adults in freshwater fishes. Adult specimens have been reported inhabiting the stomach or intestine of freshwater hosts such as *Astyanax abramis, Trichomycterus spegazzinii, Brycon orbignyanus, Pimelodus albicans, Pimelodus maculatus* and *Corydoras paleatus* (Ramallo and Ailán-Choke, 2022).

However, the complete life cycles of many Neotropical *Spirocamallanus* species remain poorly understood. Available evidence suggests that these nematodes follow the typical indirect camallanid cycle, in which aquatic copepods such as *Mesocyclops* spp. and *Diaptomus* spp. act as intermediate hosts and fishes serve as definitive hosts. Larvae released into the water are ingested by copepods, develop to infective stages, and are subsequently transmitted to fishes through trophic interactions. This pathway has been proposed for species such as *Procamallanus* (*Spirocamallanus*) *inopinatus* and experimentally supported for *P.* (*S.*) *hilarii* (Neves et al., 2020). Omnivorous feeding habits may increase exposure to infected copepods and other intermediate food items, and omnivorous fishes often harbour higher parasite diversity due to their broad trophic interactions (Jacobson et al., 2024; Negreiros and Tavares-Dias, 2024).

The presence of *Contracaecum*, associated with bird–fish trophic pathways, together with *Spirocamallanus*, maintained through fish–invertebrate transmission, indicates overlapping but functionally distinct routes within the same ecological network (Neves et al., 2020; Bennett et al., 2022). In addition, camallanids have been associated with intestinal lesions and inflammatory damage in fishes from the Peruvian Amazon, suggesting potential health impacts for hosts in La Mojana as well (Sánchez et al., 2024). Similarly, *Contracaecum* larvae have been associated with tissue damage, inflammatory responses and pathological alterations in freshwater fishes, including liver alterations previously documented in fishes from La Mojana (Fuentes-Lopez et al., 2025).

Patterns of host association recovered from the multilocus analyses indicate that *Contracaecum* larvae in La Mojana combine broad host use with partial lineage structuring. ITS2 sequences placed specimens from multiple fish species within the *C. jorgei* clade, suggesting low larval host specificity and shared transmission networks involving piscivorous birds. In contrast, and considering much fewer sequences are available for these markers, *rrnS* and 18S markers revealed partial clustering by host species, particularly among predatory fishes, which may reflect differences in trophic exposure, local population structure or early host-associated divergence. Overall, these findings suggest that host association in *Contracaecum* is shaped by a combination of ecological generalism and finer-scale transmission structuring within the floodplain fish assemblage, as reported in other anisakid systems characterized by broad host use and variable host specificity (Vuić et al., 2022; Garbin et al., 2024b).

Parasites can interact with contaminants and other ecological pressures to influence host health and condition (Gorgoglione et al., 2020; Sures and Nachev, 2022). In La Mojana, freshwater fish represent a primary source of animal protein and constitute the basis of subsistence fisheries for local communities (Baleta-Anaya et al., 2022). The detection of anisakid larvae in edible fish raises public health considerations, even in freshwater systems where anisakidosis is less frequently reported than in marine environments. *Contracaecum* is particularly relevant, as these nematodes may represent a zoonotic risk when infected fish are consumed raw or insufficiently cooked (Kaba et al., 2024; Shamsi, 2025). Therefore, the presence of *Contracaecum* larvae in locally consumed fish warrants attention from both ecological and food safety perspectives (Castellanos-Garzón et al., 2020). Moreover, visible parasite burdens can influence consumer perception, market acceptance and the economic value of fish products. In subsistence-based economies, reductions in marketability may directly impact household income and food security. In the present study, *Contracaecum* sp. were frequently recovered in relatively high numbers from visceral organs of several fish hosts, a feature that may reduce consumer acceptance due to the visible appearance of infection.

In conclusion, the present study provides the first integrative molecular and morphological evidence of anisakid and camallanid nematodes infecting freshwater fishes from La Mojana. Combined morphological and molecular evidence supported the identification of *Contracaecum jorgei*, a second *Contracaecum* lineage related to the *C. multipapillatum* complex, and *Spirocamallanus* sp. closely related to *S. hilarii*, the latter representing a new geographic record of *Spirocamallanus* sp. for the department of Sucre. By combining morphological and multilocus genetic data, our findings clarify host–parasite association patterns, demonstrate trophic structuring of parasite assemblages and highlight both ecological complexity and potential sanitary implications in a tropical wetland system that remains understudied.

## Supporting information

10.1017/S003118202610273X.sm001 Fuentes-Lopez et al. supplementary material Fuentes-Lopez et al. supplementary material

## Supplementary material

The supplementary material for this article can be found at https://doi.org/10.1017/S003118202610273X.

## Table 1 Long description

Columns list common name, scientific name, family, feeding habit, sample size, trophic level, mean total length in cm, mean total weight in g, number of parasites, mean abundance plus or minus SEM, and mean intensity plus or minus SEM for 10 fish species. All species have sample size 5, and feeding habit is carnivore piscivore for nine species and omnivores for Comelon. Trophic level ranges from 2.6 for Comelon to 4.5 for Moncholo and Pacora. Mean total length ranges from 20.2 cm for Cachegua to 70.4 cm for Mayupa, and mean total weight ranges from 104.0 g for Cachegua to 782.4 g for Bagre rayado. Number of parasites by species is Moncholo 21, Pacora 5, Bagre blanquillo 77, Bagre rayado 88, Chango 13, Barbul 5, Cachegua 17, Mojarra Amarilla 15, Mayupa 37, and Comelon 11 with an asterisk marker. Mean abundance plus or minus SEM is Moncholo 4.2 plus or minus 1.4, Pacora 1.0 plus or minus 0.0, Bagre blanquillo 15.4 plus or minus 10.1, Bagre rayado 17.6 plus or minus 4.3, Chango 2.6 plus or minus 0.7, Barbul 1.0 plus or minus 0.3, Cachegua 3.4 plus or minus 1.1, Mojarra Amarilla 3.0 plus or minus 1.2, Mayupa 7.4 plus or minus 3.7, and Comelon 2.2 plus or minus 0.7. Mean intensity plus or minus SEM is Moncholo 5.3 plus or minus 1.1, Pacora 1.0 plus or minus 0.0, Bagre blanquillo 15.4 plus or minus 10.1, Bagre rayado 17.6 plus or minus 4.3, Chango 3.3 plus or minus 0.3, Barbul 1.3 plus or minus 0.3, Cachegua 4.3 plus or minus 1.0, Mojarra Amarilla 3.8 plus or minus 1.3, Mayupa 7.4 plus or minus 3.7, and Comelon 2.8 plus or minus 0.6.

Navigate back to Table 1.

## Figure 3 Long description

Two Bayesian inference phylogenetic trees are presented, labeled A and B. Tree A uses the ITS2 genetic marker, while tree B uses the mitochondrial rrnS marker. In both trees, specimens analyzed in the study are highlighted in blue, with their host species indicated next to the sequences. Bayesian posterior probabilities are marked at the nodes, with different symbols representing ranges from 0.6 to 1.0. In tree A, the scale bar represents 0.07 nucleotide substitutions per site and in tree B, it represents 0.04. Tree A shows a detailed branching pattern with various fish species, including C. australe and C. pagrosomi, among others. Tree B includes species such as C. multipapillatum and C. osculatum, with a similar branching structure. Both trees illustrate the genetic similarity among specimens collected from different host species, supported by high Bayesian posterior probability values.

Navigate back to Figure 3.

## Figure 4 Long description

Two phylogenetic trees labeled A and B represent Bayesian inference phylogenetic trees reconstructed using nuclear 18S rRNA and 28S rRNA genetic markers, respectively. In tree A, the 18S rRNA phylogeny is shown with a horizontal orientation, starting from the top left and extending to the right. The tree includes several branches with nodes indicating Bayesian posterior probabilities. Specimens analyzed in the study are highlighted in blue, with their host species indicated next to the sequences. The scale bar represents the number of nucleotide substitutions per site, marked as 0.003. The tree places specimens recovered from various hosts within a well-supported clade corresponding to Contracaecum sp., with sequence identities ranging from 95 to 100 percent. The sequences cluster closely with C. multipapillatum and are positioned near other congeners. In tree B, the 28S rRNA phylogeny is similarly oriented horizontally, with branches and nodes indicating Bayesian posterior probabilities. The scale bar is marked as 0.01. The specimen recovered from M. muyscorum is placed within a well-supported clade of Contracaecum sp., showing more than 95 percent identity with C. multipapillatum. The trees illustrate genetic relationships and divergence among the specimens and related lineages.

Navigate back to Figure 4.

## Figure 5 Long description

The diagram is a phylogenetic tree reconstructed using the nuclear 18S rRNA marker. It shows the evolutionary relationships among various specimens, with Bayesian posterior probabilities indicated at the nodes. The tree is oriented vertically, with branches extending from top to bottom. Specimens analyzed in the study are highlighted in blue, with their host species indicated next to the sequences. The scale bar represents the number of nucleotide substitutions per site. The tree includes sequences from different geographical locations, such as Brazil, Egypt, Argentina and New Caledonia. Bayesian posterior probabilities are represented by different shapes at the nodes: squares for probabilities between 0.95 and 1.0, circles for 0.8 to 0.94 and triangles for 0.5 to 0.79. The tree illustrates genetic similarity among specimens collected from different host species, with sequences grouped together with high Bayesian posterior probability support.

Navigate back to Figure 5.
