# Supplementary material for: Morphological and molecular characterization of nematodes in a freshwater fish community from La Mojana, Sucre, Colombia
Source: Parasitology. 2026 Sep 8;153(9):1206–17. doi: 10.1017/S003118202610273X (PMC13735943; doi:10.1017/S003118202610273X)
Supplement: Supplementary file 1 [file S003118202610273Xsup001.docx]

**Morphological and molecular characterization of nematodes in a freshwater fish community from La Mojana, Sucre (Colombia)**

Katerin Fuentes-Lopez^1,2^, Karina Caballero-Gallardo^1,2^, Robert Poulin^3*^, Jerusha Bennett^3^

1 Environmental and Computational Chemistry Group. School of Pharmaceutical Sciences, Zaragocilla Campus, University of Cartagena, Cartagena 130014, Colombia

2 Functional Toxicology Group. School of Pharmaceutical Sciences, Zaragocilla Campus, University of Cartagena, Cartagena 130014, Colombia

3 Department of Zoology, University of Otago, Dunedin 9054, New Zealand

*. Corresponding Author:

E-mail: [robert.poulin@otago.ac.nz](mailto:robert.poulin@otago.ac.nz)

**Content**

I. **Supplementary Table S1**. GenBank accession numbers and metadata for nematode sequences generated in this study, including taxonomic identification, gene marker, host species, isolation source, life stage, collection locality, and collection date.
II. **Supplementary Figure S1**. Third-stage larvae (L3) of *Contracaecum* sp. recovered from the mesentery of *Pseudoplatystoma magdaleniatum* (A) and *Trachelyopterus insignis* (B). Isolated third-stage larva (L3) of *Contracaecum* sp (C).
III. **Supplementary Figure S2**. Composite micrograph of *Contracaecum* sp. reconstructed from multiple overlapping images acquired at the same magnification (scale bar in each panel = 500 µm). The assembled image allows visualization of the complete larval morphology, including the anterior lips and posterior mucron, while preserving fine structural details.
IV. **Supplementary Figure S3**. Light micrographs of female specimens of *Spirocamallanus* recovered from the freshwater fish *M. muyscorum*. Scale bars = 200 µm. (A) Anterior region showing the buccal capsule (b), muscular esophagus (m), and glandular esophagus (g). (B) Midbody region showing first-stage larvae (l) within the uterus. (C) Posterior region of a gravid female showing first-stage larvae (l) and the tail (t).
V. **Supplementary Table S2**. Estimates of Evolutionary Divergence between Sequences (p-distance) based on the 28S marker.

**Supplementary Table S1.** GenBank accession numbers and associated metadata for nematode sequences generated in this study, including taxonomic identification, molecular marker, host species, isolation source, and life stage, all collected from La Mojana, Sucre, Colombia in April 2025.

| **Sample ID** | **Organism** | **Gene** | **Host** | **Isolation source** | **Life stage** | **GenBank accession numbers** |
| --- | --- | --- | --- | --- | --- | --- |
| M16 | *Contracaecum jorgei* | ITS2 | *Megaleporinus muyscorum* | Mesenteries | Larva | PZ394368 |
| M30 | *Contracaecum jorgei* | ITS2 | *Sorubim cuspicaudus* | Mesenteries | Larva | PZ394369 |
| M31 | *Contracaecum jorgei* | ITS2 | *Pseudoplatystoma magdaleniatum* | Mesenteries | Larva | PZ394370 |
| M32 | *Contracaecum jorgei* | ITS2 | *Sternopygus macrurus* | Mesenteries | Larva | PZ394371 |
| M37 | *Contracaecum jorgei* | ITS2 | *Sorubim cuspicaudus* | Mesenteries | Larva | PZ394372 |
| M39 | *Contracaecum jorgei* | ITS2 | *Polydactylus virginicus* | Mesenteries | Larva | PZ394373 |
| M36 | *Contracaecum jorgei* | ITS2 | *Caquetaia kraussii* | Mesenteries | Larva | PZ394374 |
| M15 | *Contracaecum jorgei* | ITS2 | *Megaleporinus muyscorum* | Mesenteries | Larva | PZ394375 |
| M1 | *Contracaecum jorgei* | ITS2 | *Hoplias malabaricus* | Mesenteries | Larva | PZ394376 |
| M11 | *Contracaecum jorgei* | ITS2 | *Cynopotamus magdalenae* | Mesenteries | Larva | PZ394377 |
| M6 | *Contracaecum jorgei* | ITS2 | *Pseudoplatystoma magdaleniatum* | Mesenteries | Larva | PZ394378 |
| M17 | *Contracaecum jorgei* | ITS2 | *Plagioscion surinamensis* | Mesenteries | Larva | PZ394379 |
| M43 | *Contracaecum jorgei* | ITS2 | *Megaleporinus muyscorum* | Mesenteries | Larva | PZ394380 |
| M25 | *Contracaecum jorgei* | ITS2 | *Caquetaia kraussii* | Mesenteries | Larva | PZ394381 |
| M35 | *Contracaecum jorgei* | ITS2 | *Sternopygus macrurus* | Mesenteries | Larva | PZ394382 |
| M38 | *Contracaecum jorgei* | ITS2 | *Megaleporinus muyscorum* | Mesenteries | Larva | PZ394383 |
| M27 | *Contracaecum jorgei* | ITS2 | *Pseudoplatystoma magdaleniatum* | Mesenteries | Larva | PZ394384 |
| M9 | *Contracaecum jorgei* | ITS2 | *Sorubim cuspicaudus* | Mesenteries | Larva | PZ394385 |
| M8 | *Contracaecum jorgei* | ITS2 | *Pseudoplatystoma magdaleniatum* | Mesenteries | Larva | PZ394386 |
| M19 | *Contracaecum jorgei* | ITS2 | *Trachelyopterus insignis* | Mesenteries | Larva | PZ394387 |
| M20 | *Contracaecum jorgei* | ITS2 | *Trachelyopterus insignis* | Mesenteries | Larva | PZ394388 |
| M28 | *Contracaecum jorgei* | ITS2 | *Sorubim cuspicaudus* | Mesenteries | Larva | PZ394389 |
| M29 | *Contracaecum jorgei* | ITS2 | *Sorubim cuspicaudus* | Mesenteries | Larva | PZ394390 |
| M2 | *Contracaecum jorgei* | ITS2 | *Hoplias malabaricus* | Mesenteries | Larva | PZ394391 |
| M4 | *Contracaecum jorgei* | ITS2 | *Hoplias malabaricus* | Mesenteries | Larva | PZ394392 |
| M5 | *Contracaecum jorgei* | ITS2 | *Hoplias malabaricus* | Mesenteries | Larva | PZ394393 |
| M18 | *Contracaecum jorgei* | ITS2 | *Trachelyopterus insignis* | Mesenteries | Larva | PZ394394 |
| M22 | *Contracaecum jorgei* | ITS2 | *Trachelyopterus insignis* | Mesenteries | Larva | PZ394395 |
| M40 | *Contracaecum jorgei* | ITS2 | *Plagioscion surinamensis* | Mesenteries | Larva | PZ394396 |
| M33 | *Contracaecum jorgei* | ITS2 | *Sternopygus macrurus* | Mesenteries | Larva | PZ394397 |
| M23 | *Contracaecum jorgei* | ITS2 | *Caquetaia kraussii* | Mesenteries | Larva | PZ394398 |
| M26 | *Contracaecum jorgei* | ITS2 | *Caquetaia kraussii* | Mesenteries | Larva | PZ394399 |
| M13 | *Contracaecum jorgei* | ITS2 | *Cynopotamus magdalenae* | Mesenteries | Larva | PZ394400 |
| M7 | *Contracaecum jorgei* | ITS2 | *Pseudoplatystoma magdaleniatum* | Mesenteries | Larva | PZ394401 |
| M3 | *Contracaecum jorgei* | ITS2 | *Plagioscion surinamensis* | Mesenteries | Larva | PZ394402 |
| M34 | *Contracaecum jorgei* | ITS2 | *Sternopygus macrurus* | Mesenteries | Larva | PZ394403 |
| M41 | *Contracaecum jorgei* | rrnS | *Polydactylus virginicus* | Mesenteries | Larva | PZ410428 |
| M42 | *Contracaecum jorgei* | rrnS | *Hoplias malabaricus* | Mesenteries | Larva | PZ410429 |
| M7 | *Contracaecum* sp. | 18S | *Pseudoplatystoma magdaleniatum* | Mesenteries | Larva | PZ394415 |
| M9 | *Contracaecum* sp. | 18S | *Sorubim cuspicaudus* | Mesenteries | Larva | PZ394416 |
| M16 | *Contracaecum* sp. | 18S | *Megaleporinus muyscorum* | Mesenteries | Larva | PZ394417 |
| M32 | *Contracaecum* sp. | 18S | *Sternopygus macrurus* | Mesenteries | Larva | PZ394418 |
| M16 | *Contracaecum* sp. | 28S | *Megaleporinus muyscorum* | Mesenteries | Larva | PZ403467 |
| M10 | *Spirocamallanus* sp. | 18S | *Megaleporinus muyscorum* | Intestine | Adult | PZ394420 |
| M43 | *Spirocamallanus* sp. | 18S | *Megaleporinus muyscorum* | Intestine | Adult | PZ394421 |
| M10 | *Procamallanus* sp. | 28S | *Megaleporinus muyscorum* | Intestine | Adult | PZ403468 |
| M43 | *Procamallanus* sp. | 28S | *Megaleporinus muyscorum* | Intestine | Adult | PZ403469 |

**Figure S1.** Third-stage larvae (L3) of *Contracaecum* sp. recovered from the mesentery of *Pseudoplatystoma magdaleniatum* (A) and *Trachelyopterus insignis* (B). Isolated third-stage larva (L3) of *Contracaecum* sp (C).

**Figure S2.** Composite micrograph of *Contracaecum* sp. reconstructed from multiple overlapping images acquired at the same magnification (scale bar in each panel = 500 µm). The assembled image allows visualization of the complete larval morphology, including the anterior lips and posterior mucron, while preserving fine structural details.

**Figure S3.** Light micrographs of female specimens of *Spirocamallanus* recovered from the freshwater fish *M. muyscorum*. Scale bars = 200 µm. (A) Anterior region showing the buccal capsule (b), muscular esophagus (m), and glandular esophagus (g). (B) Midbody region showing first-stage larvae (l) within the uterus. (C) Posterior region of a gravid female showing first-stage larvae (l) and the tail (t).

**Table S2.** Estimates of Evolutionary Divergence between Sequences (p-distance) based on the 28S marker.

|  | **M10** | **M43** | **Procamallanus neocaballeroi (MT582734)** | **Procamallanus neocaballeroi (MT582728)** | **Spirocamallanus hilarii (MW930866)** | **Procamallanus pintoi (MW930867)** | **Procamallanus rebecae (MW485586)** | **Procamallanus barlowi (MW485594)** | **Spirocamallanus inopinatus (MT901636)** | **Spirocamallanus inopinatus (MT901638)** |
| --- | --- | --- | --- | --- | --- | --- | --- | --- | --- | --- |
| **M10** |  |  |  |  |  |  |  |  |  |  |
| **M43** | 0.0000 |  |  |  |  |  |  |  |  |  |
| **Procamallanus neocaballeroi (MT582734)** | 0.0254 | 0.0256 |  |  |  |  |  |  |  |  |
| **Procamallanus neocaballeroi (MT582728)** | 0.0254 | 0.0256 | 0.0021 |  |  |  |  |  |  |  |
| **Spirocamallanus hilarii (MW930866)** | 0.0883 | 0.0871 | 0.0668 | 0.0668 |  |  |  |  |  |  |
| **Procamallanus pintoi (MW930867)** | 0.2007 | 0.2018 | 0.1550 | 0.1550 | 0.1570 |  |  |  |  |  |
| **Procamallanus rebecae (MW485586)** | 0.2212 | 0.2214 | 0.1529 | 0.1541 | 0.1733 | 0.1660 |  |  |  |  |
| **Procamallanus barlowi (MW485594)** | 0.1992 | 0.1992 | 0.1398 | 0.1421 | 0.1621 | 0.1715 | 0.1081 |  |  |  |
| **Spirocamallanus inopinatus (MT901636)** | 0.1891 | 0.1886 | 0.1575 | 0.1575 | 0.1521 | 0.1508 | 0.1740 | 0.1775 |  |  |
| **Spirocamallanus inopinatus (MT901638)** | 0.1835 | 0.1830 | 0.1526 | 0.1526 | 0.1484 | 0.1396 | 0.1778 | 0.1796 | 0.0171 |  |

The number of base differences per site between sequences is shown. The analytical procedure encompassed 10 nucleotide sequences. The pairwise deletion option was applied to all ambiguous positions for each sequence pair resulting in a final data set comprising 1.058 positions. Evolutionary analyses were conducted in MEGA v12
